# Energy Shifts in Predissociating Levels of Diatomic Molecules: The Case of N_2_ (C ″^5^Π_*u*_) and N_2_ (1 ^7^Σ_*u*_^+^) Interacting States

**DOI:** 10.1021/acsomega.5c03821

**Published:** 2025-07-09

**Authors:** Laiz R. Ventura, Ramon S. da Silva, Sergio R. Souza, Jayr Amorim, Carlos E. Fellows

**Affiliations:** † Departamento de Física, Instituto Tecnológico de Aeronáutica, 12228-900 São José dos Campos, Brazil; ‡ Departmento de Física, Instituto de Ciências Exatas—ICEx, 28110 Universidade Federal Fluminense, Campus do Aterrado, Volta Redonda, Rio de Janeiro 27213-45, Brazil; § Departamento de Física, Universidade Federal de Juiz de Fora, Juiz de Fora, State of Minas Gerais 36036-900, Brazil; ∥ Instituto de Física, Universidade Federal do Rio de Janeiro, Rio de Janeiro, Rio de Janeiro 21941-909, Brazil

## Abstract

This work presents a perturbative calculation methodology for evaluating the energy shifts and broadening of vibrational energy levels, caused by interactions between bound and unbound dissociative electronic states. The method is validated against previously semiclassical analyzed cases, demonstrating remarkable consistency. We successfully applied this approach to the N_2_ molecule, which exhibits a strong spin–orbit interaction between the bound C ″^5^Π_*u*_ and the repulsive 1 ^7^Σ_*u*_^+^ electronic states, around 36 cm^–1^. This interaction constitutes an major pathway for N­(^2^D) production, important in both excitation and quenching in plasma afterglows. As a result, the maximum absolute shift of 0.15 cm^–1^ was found for the C ″^5^Π_*u*_ (*v* = 7) and maximum broadening of 0.45 cm^–1^ was calculated for *v* = 8, demonstrating significant perturbation of the C ″^5^Π_*u*_ by the 1 ^7^Σ_*u*_^+^ state. The results obtained were compared with direct calculations of the predissociation rates of the C ″^5^Π_*u*_ bound state, showing very good agreement.

## Graphical abstract

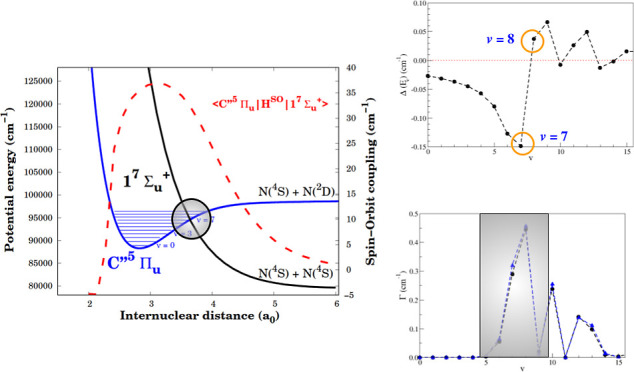

## Introduction

Perturbations between discrete vibrational levels belonging to two bound electronic states occur relatively frequently.^1,2^ However, the overlapping energy levels of a molecule with a dissociative continuum give rise to a predissociation phenomenon, which is less frequently observed.^3–6^ Herzberg^7^ previously classified these effects into three categories, with type I relating bound and unbound states, being the most significant in the case of diatomic molecules. As observed theoretically by Fano^8^ and experimentally by Barrow et al.,^9^ an isolated discrete level that interacts with an unbound (dissociative) state undergoes a broadening and a shift in energy. An accurate estimate of the width of the broadening can be obtained once the wave functions of the discrete level and the resonant continuous level are determined, as demonstrated by Murrell and Taylor,^10^ and by Czarny, Felenbok and Lefebvre-Brion.^11^ The direct numerical estimate of the shift has been performed in a few cases^11–14^ albeit in an approximate way, using model potentials to describe the interacting states, as carried out by Atabek and Lefebvre^12^ or through ab initio calculations, such as the work conducted by Julienne and Krauss.^13^ Other methods, such as semiclassical approximations, have been used, as in the work of Child^15^ and Child and Lefebvre.^16^ However, solving the coupled equations is computationally intensive while the solution using semiclassical methods is complex and nontrivial.

In this work, we present an application of the perturbative solution proposed by Fano,^8^ applied to the interaction between the bound N_2_ (C ″^5^Π_*u*_) state and the repulsive N_2_ (1 ^7^Σ_*u*_^+^). The spin–orbit coupling (SOC) between these states is relatively strong, making them attractive for use in kinetic models. According to Wu et al.,^17^ this interaction plays a crucial role in both electronic excitation and quenching processes, exhibiting rate coefficients comparable to those of the N­(^4^S) + N_2_ → N­(^2^D) + N_2_ reaction. The results presented in the following sections demonstrate promising potential for modeling these bound-unbound state interactions, suggesting new opportunities for investigating similar interacting level systems.

The choice of the N_2_ molecule and the electronic states C ″^5^Π_*u*_ and 1 ^7^Σ_*u*_^+^ is a natural consequence of the work recently carried out by Ventura et al.,^18^ where it was observed that their interaction significantly modifies the lifetime of the C ″^5^Π_*u*_ state.

## Energy Levels Shift Evaluations

The unperturbed discrete energy spectrum *E*_*v*_ of the Hamiltonian *H*, describing the vibrational states of the diatomic molecule, is obtained by solving the Schrodinger equation considering a potential *V*_b_, such that

1 $$\left\langle \psi_{v^{\prime }}\left|H\right|\psi_{v}\right\rangle =E_{v}\delta_{v,v^{\prime }}$$

where |ψ_v_⟩ is the wave function associated with the *v*-th vibrational state and δ_v,v′_ represents the Kronecker delta function. The shift in the energy *E*_v_ is given by

2 $$E_{v}^{\prime }=E_{v}+\Delta \left(E_{v}\right)$$

due to the coupling with continuum states |ϕ_E_⟩ of energy *E* of the Hamiltonian, is calculated through the perturbative treatment developed by Fano,^8^ from which one writes

3 $$\Delta \left(E_{v}\right)=P\int dE^{\prime }\frac{{\left|V_{E^{\prime }}^{v}\right|}^{2}}{E_{v}-E^{\prime }}$$

where P indicates “the principal part of” and *V*_E_^v^ = ⟨ϕ_E_|*H*|ψ_v_⟩ is the potential coupling between bound and continuum states. The latter are calculated using a repulsive potential, *V*_u_, and are normalized such that

4 $$\left\langle \phi_{E^{\prime }}\left|H\right|\phi_{E}\right\rangle =E\delta \left(E-E^{\prime }\right)$$

The Dirac delta function δ­(*E* – *E*′) ensures that |ϕ_E_⟩ is normalized per unit energy, so that ${\left|V_{E^{\prime }}^{v}\right|}^{2}$ has the dimensionality of *E*.

As a first step, in order to verify the accuracy of our calculations, a comparison with the data reported in the literature is performed. To do this, a simple model already proposed by Child and Lefebvre^16^ is used. In it, the attractive potential *V*_b_ is given by a Morse potential

5 $$V_{b}\left(r\right)=D{\left[1-\exp\left(-\beta \left(r-r_{e}\right)\right)\right]}^{2}$$

and the repulsive potential *V*_u_ is represented by

6 $$V_{u}\left(r\right)=A\;\exp\left[-\alpha \left(r-r_{c}\right)\right]+B$$

The set of parameters used by Child and Lefebvre^16^ in their calculations, reads: *A* = 18,154.95 cm^–1^, *D* = 15,000 cm^–1^, *B* = – 8000 cm^–1^, *r*_e_ = 1.6 Å, *r*_C_ = 248 Å, α = 2.2039 Å^–1^ and β = 1.9685 Å^–1^. The electronic interaction, associated with *V*_E_^v^, is set to β = 100 cm^–1^ and the value of the reduced mass μ is 8 Da. The potential energy curves are defined so that the repulsive and attractive ones cross each other at *r*_C_, which is the turning point to the right of the *v* = 18 level of the Morse potential. Our results are shown in Figure 1, along with those obtained by Child and Lefebvre^16^ who solved the corresponding coupled Schrödinger equations. As can be seen, the results are in good agreement. Figure 1 illustrates excellent concordance between the results obtained from both methodologies.

**Figure 1:**
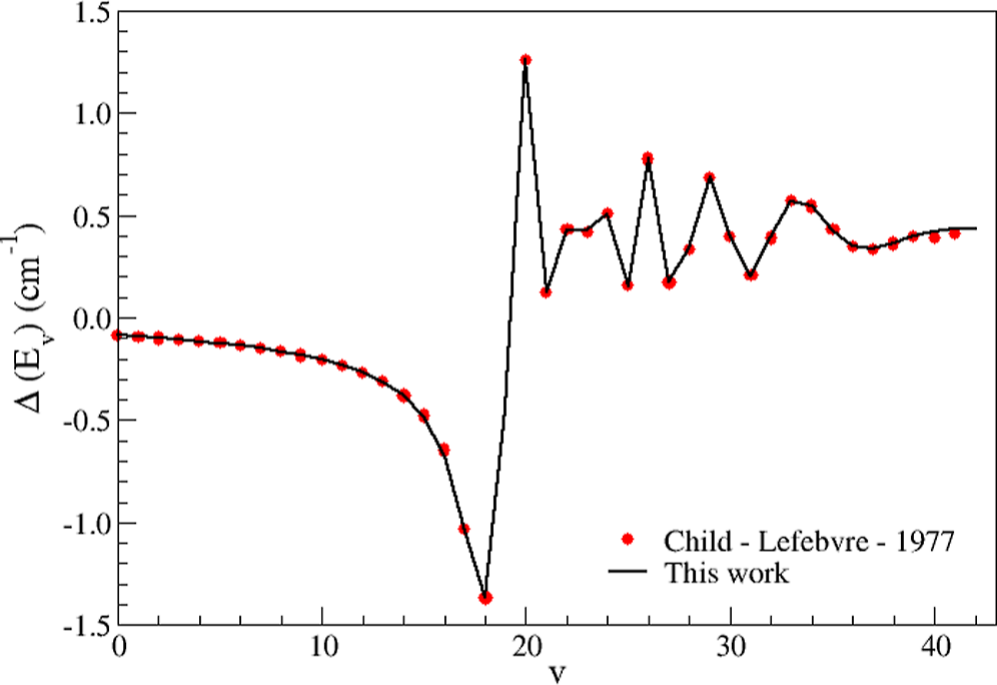
Comparison between the results obtained by Child and Lefebvre^16^ and those obtained in the present article.

## The N_2_ C ″^5^Π_*u*_ and 1 ^7^Σ_*u*_^+^ Interaction Case

The most relevant potential energy curves for this work were calculated by Hochlaf et al.^19^ and da Silva et al.^20^ In these two works, potential energy curves were obtained for the triplet, quintet and septet states. Hochlaf et al.^19^ were the first to present theoretical calculations of the spin–orbit interaction between triplet and quintet states, as well as between quintet and septet states in the N_2_ molecule. Later, new calculations were performed by Ventura et al.^18^ with new values for the spin–orbit coupling for the C ″^5^Π_*u*_ and 1 ^7^Σ_*u*_^+^ electronic states.

We can now turn our attention to the energy shifts Δ­(*E*_v_) of the bound vibrational levels *E*_v_ of C ″^5^Π_*u*_ due to the interaction with the repulsive state 1 ^7^Σ_*u*_^+^ of the N_2_ molecule. The potential energy and spin–orbit interaction curves used in this work for the C ″^5^Π_*u*_ and 1 ^7^Σ_*u*_^+^ states were taken from da Silva et al.^20^ and Ventura et al.^18^ In summary, these authors reported high-level multireference configuration interaction (MRCI) calculations combined with a larger Dunning basis set (AV5Z). This choice was made because this method has successfully reproduced experimental results with an acceptable degree of reliability. For convenience, these results, up to vibrational level *v* = 15, are shown in Figure 2.

**Figure 2:**
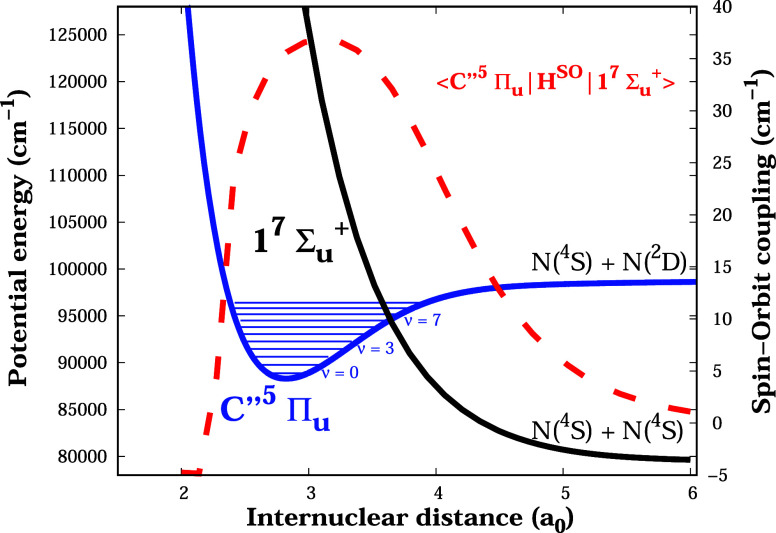
Figure shows the potential energy curves of the electronic states C ″^5^Π_*u*_ and 1 ^7^Σ_*u*_^+^ (solid lines). Also shown is the spin–orbit interaction curve between the states (dashed line) as a function of the internuclear distance.

It should be noted that Figure 2 illustrates that the maximum spin–orbit coupling (around 36 cm^–1^) is at the internuclear distance where energy curves of the N_2_ C ″^5^Π_*u*_ and 1 ^7^Σ_*u*_^+^ states intersect. The vibrational energy values *E*_v_ with respect to the minimum of the potential curve of the electronic state C ″^5^Π_*u*_ at 87,703.5 cm^–1^, obtained as described above, and the corresponding shifts Δ­(*E*_v_) given by eq 3, are listed in Table 1. The latter are also plotted in Figure 3.

**Table 1:** Energy Levels, with Respect to the Minimum of the Potential Curve of the Electronic State C ″^5^Π_*u*_ (87,703.5 cm^–1^), Given by eq 1 and Corresponding Displacements Calculated by eq 3

*v*	*E*_v_ (cm^–1^)	Δ(*E*_v_) (cm^–1^)	*v*	*E*_v_ (cm^–1^)	Δ(*E*_v_) (cm^–1^)
0	453.87	–0.0267	8	6757.08	0.0372
1	1342.37	–0.0312	9	7390.77	0.0663
2	2204.67	–0.0370	10	7979.18	–0.0075
3	3040.02	–0.0450	11	8516.90	0.0263
4	3847.27	–0.0571	12	8997.28	0.0494
5	4624.85	–0.0800	13	9412.07	–0.0127
6	5370.77	–0.1270	14	9751.28	–0.0019
7	6082.52	–0.1490	15	10,005.00	0.0156

**Figure 3:**
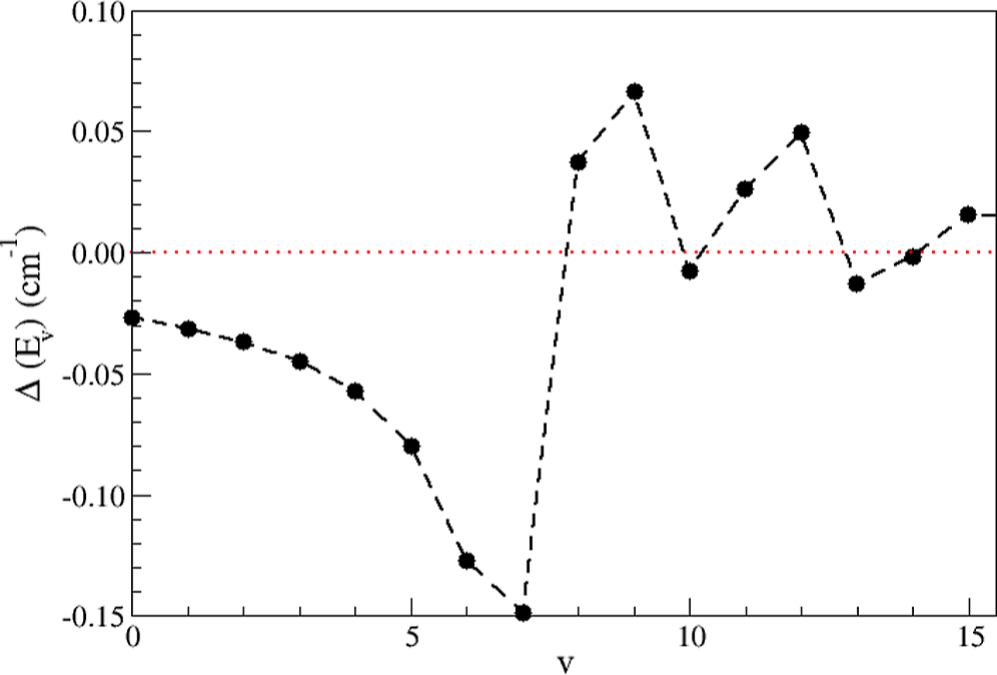
Energy shift in the lowest vibrational levels of the C ″^5^Π_*u*_ electronic state due to interaction with the 1 ^7^Σ_*u*_^+^ electronic state, as a function of the vibrational quantum number.

### Predissociation and Lifetimes

It can be seen from Table 1 and Figure 3 that the energy shifts of the electronic state C ″^5^Π_*u*_ become more significant starting at the vibrational level v = 5, where the spin–orbit interaction with the dissociative state 1 ^7^Σ_*u*_^+^ reaches the highest values, as shown in Figure 2. The maximum value of the energy shift is reached at v = 7. From this vibrational level, the variations in the energy deviation become oscillatory up to the value of v = 14, and then decay monotonically. This behavior is due to the interaction between the bound levels of the C ″^5^Π_*u*_ electronic state and the continuum of the 1 ^7^Σ_*u*_^+^ dissociative state.

A measure of this interaction is provided by the Franck–Condon factor ⟨ϕ_E_||ψ_v_⟩, which is closely related to the interaction matrix element *V*_*E*_^v^. Figure 4 displays the values of |⟨ϕ_E_||ψ_v_⟩|^2^ as a function of energy for the vibrational quantum numbers v = 7 and v = 9.

**Figure 4:**
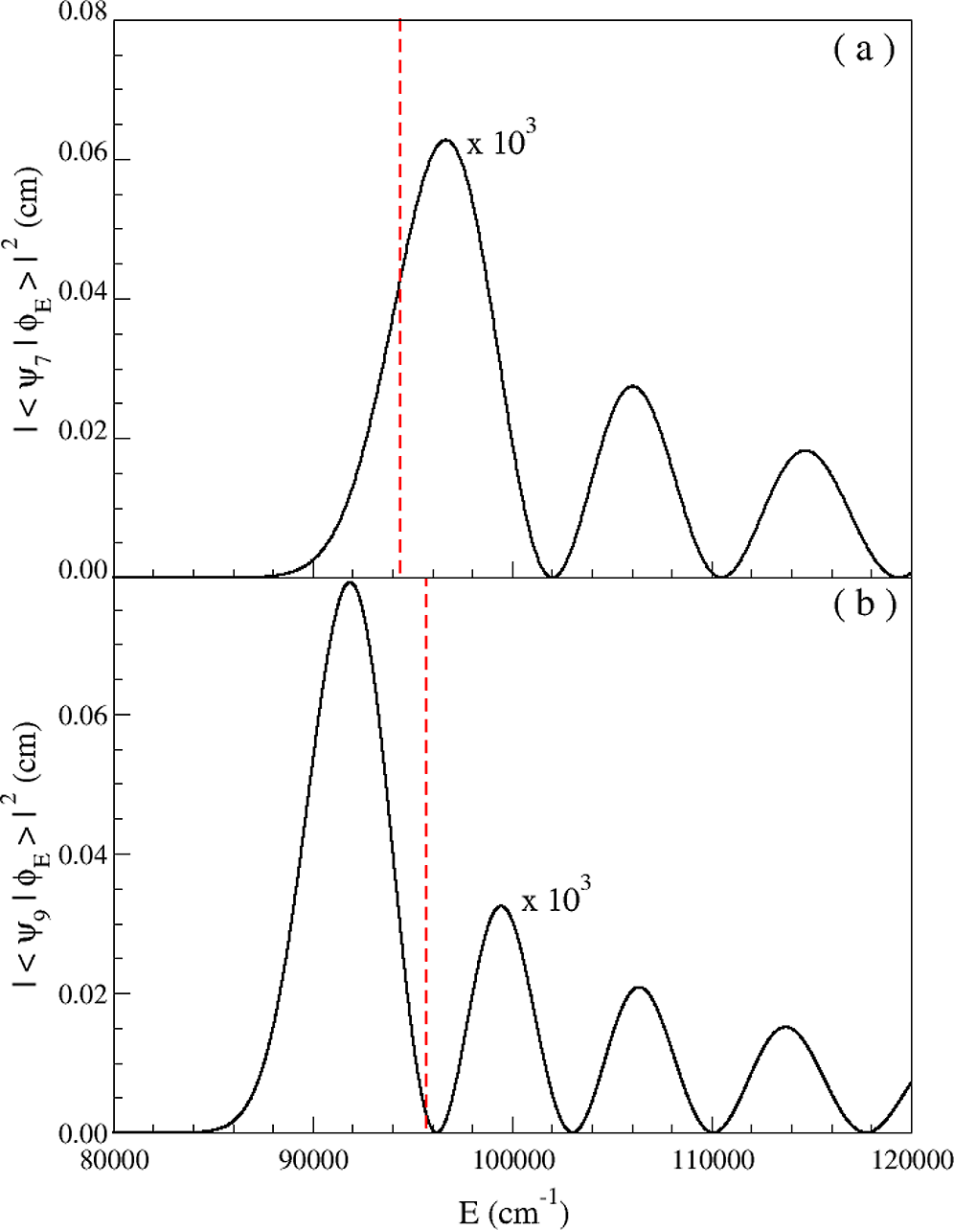
Square of the Franck–Condon factors as a function of the energy for the (a) v = 7 vibrational level, (b) v = 9 vibrational level. The dashed lines indicates the position of the discrete levels.

In order to provide a sense of the positions of the discrete levels, a dashed line is included in the graphs for this purpose. These quantities exhibit rapid oscillations strongly correlated with the energy. The widths of the vibrational levels, given by $\Gamma =2\pi {\left|V_{E^{\prime }}^{v}\right|}^{2}$ ,^8^ are shown in Figure 5 as a function of the vibrational quantum number.

**Figure 5:**
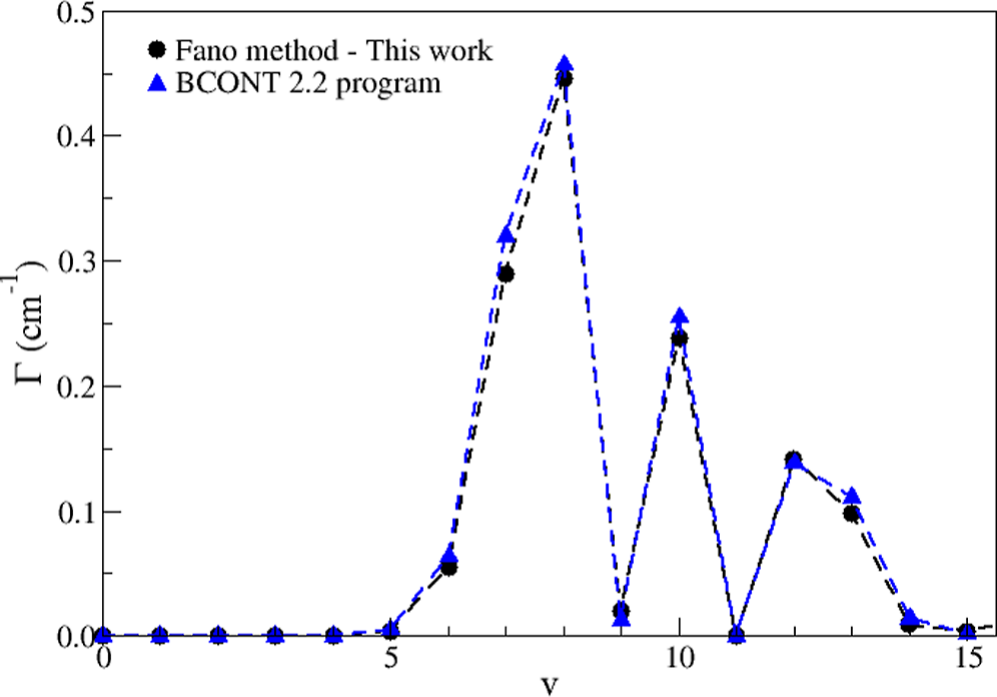
Level width as a function of the vibrational quantum number *v*.

In order to analyze the interaction between the two electronic states in greater depth, an alternative procedure was adopted in which the lifetimes were directly computed. As a first step, the lifetimes of the vibrational levels of the electronic state C ″^5^Π_*u*_ were calculated from the C ″^5^Π_*u*_ → A ^′5^Σ_g_^+^ vibronic transition employing the LEVEL 16 program.^21^ The potential energy curves of these electronic states and the transition dipole moment reported by da Silva et al.^20^ were used in the LEVEL 16 input file for the calculations. The results obtained for the radiative lifetimes (τ_rad_) for the lowest 16 vibrational levels of the C ″^5^Π_*u*_ (v = 0–15) electronic state are presented in the second column (τ_rad_) of Table 2.

**Table 2:** Radiative (τ_rad_) and Predissociated (τ_pred_) Lifetimes for the Vibrational Levels of the C ″^5^Π_*u*_ Calculated as Explained in the Text and Effective Lifetimes (τ_eff_) Calculated by Using eq 7 ^a^

*v*	τ_rad_ (ns)	τ_pred_ (ns)	τ_eff_ (ns)
0	3172.67	7.0693 × 10^10^	3172.67
1	3744.95	6.8216 × 10^7^	3744.74
2	4829.92	2.1586 × 10^5^	4724.22
3	4813.47	1.6765 × 10^3^	1243.41
4	5994.13	28.612	28.48
5	6352.97	1.0432	1.043
6	9364.68	0.0837	0.084
7	10,085.65	0.01661	0.0166
8	23,459.13	0.01162	0.0116
9	153.13 × 10^3^	0.41879	0.4188
10	1.85 × 10^6^	0.0208	0.0208
11	4.36 × 10^6^	27.389	27.389
12	5.42 × 10^6^	0.0384	0.0384
13	1.04 × 10^8^	0.0480	0.048
14	1.48 × 10^10^	0.3783	0.3783
15	8.56 × 10^10^	2.9141	2.9141

a All values in ns.

These results reveal that the radiative lifetime values remain around a few microseconds up to vibrational levels *v* = 4. Beyond this quantum number, the radiative lifetime values begin to increase, reaching up to a few milliseconds at *v* = 10 and attaining values of a few seconds for *v* = 15. This behavior is in line with the values predicted in previous work, such as da Silva et al.^20^ and Ventura et al.^18^ and reflects the fact that, as the vibrational quantum number of the higher electronic state C ″^5^Π_*u*_ increases, the Franck–Condon factors for the transition with the electronic state A ^′5^Σ_*g*_^+^ become smaller, implying a lower transition probability.

To analyze the predissociation process, the BCONT 2.2 program, developed by Le Roy and Kraemer,^22^ was employed to calculate the dissociative lifetimes of the C ″^5^Π_*u*_ electronic state due to its interaction with the 1 ^7^Σ_*u*_^+^ state. To do this, the potential energy curves calculated by da Silva et al.^20^ and the spin–orbit interaction curve reported by Ventura et al.^18^ were used. The results are shown in the third column (τ_pred_) of Table 2. The predissociative lifetime values are shown to be extremely high for values of vibrational quantum number v = 2, decreasing to a few microseconds at v = 3 and rapidly being reduced to a few tens of picoseconds for values of *v* above 6. Based on the values listed in Table 2, columns 2 and 3, assuming that both the radiative decay process and spin–orbit induced predissociation are active, the effective lifetime τ_eff_ can be calculated using the following expression

7 $$\frac{1}{\tau_{eff}}=\frac{1}{\tau_{rad}}+\frac{1}{\tau_{pred}}$$

with the corresponds results presented in the fourth column of Table 2.

## Results and Discussion

The radiative and predissociative lifetimes were obtained using the LEVEL 16 ^21^ and BCONT 2.2 ^22^ programs, respectively. As discussed above, the energy shifts and the widths of the vibrational levels Γ were calculated using the perturbative calculations. The results presented in Table 1 and Figure 3 indicate that the energy shifts become increasingly significant from v = 5, as previously mentioned. Unfortunately, all high-resolution experimental data^23–27^ lack information regarding the vibrational levels of the C ″^5^Π_*u*_ state above v = 4, for very low rotational quantum number values *J*, even for the experiments that were not carried out in a supersonic jet.^27^ Consequently, there is still no experimental data to corroborate these deviations.

Let us focus our attention on the Γ values calculated using the perturbative methods described previously and exhibited in Figure 5. One notes that the values for the broadening of the vibrational levels start to deviate from zero at v = 5, where the energy deviations become more pronounced. From then on, the Γ values increase, reaching a maximum at v = 8, and subsequently decrease to a small value at v = 9, increasing again at v = 10. It should be emphasized that Γ is linked to the average lifetime of the vibrational level through ℏ/Γ,^8^ as well as with the predissociation rate *k*_s_ calculated directly through BCONT 2.2, by the relation Γ = *k*_s_/2π*c*, where *c* is the speed of light.

Moreover, another noteworthy observation can be drawn by examining the fourth column of Table 2. The effective lifetimes (τ_eff_), calculated using eq 7 and the results obtained with the programs LEVEL 16 ^21^ and BCONT 2.2,^22^ lead to a behavior identical to that of the inverse of Γ, i.e., the lifetime values decrease from v = 5, reach a minimum at v = 8, increase at v = 9 and return, at v = 10, to a value close to that at v = 5. In order to more effectively compare the results obtained in this work with the calculations from BCONT 2.2, we have added to Figure 5 the values calculated from the relation between the dissociation rates and Γ, showing excellent agreement among the results. In other words, this behavior is consistently observed through two distinct methods.

This point can be understood by carefully examining Figure 4. Panel (a) shows that the squared Franck–Condon factor reaches a value near its maximum when the continuum energy equals the discrete energy level *E*_v_ for v = 7. This behavior is not observed in panel (b) of this Figure, which presents a similar plot for v = 9. This vibrational level intersects the squared Franck–Condon curve at values close to zero, indicating a weak interaction. This explains the increase in the effective lifetime (τ_eff_) as well as the reduction in vibrational level broadening (Γ), despite having a nonzero energy deviation Δ*E*_v_, as shown in Figure 3.

Finally, we should remember that all the treatment of spectral line broadenings and lifetime variations was carried out without taking into account rotation, Λ-doubling or fine structure effects. The methodology, as well as the computer programmes used, such as LEVEL16 ^21^ and BCONT 2.2,^22^ does not take these effects into account but has proven that a simple calculation, such as Fano’s method,^8^ predicts vibrational lifetimes in the vibrational levels with great accuracy, even showing excellent agreement with previously reported works.^18,19^ The recent paper by Mitev et al.,^28^ on the hydroxyl radical (OH), shows how these issues can be rigorously treated, including the effects of rotation, Λ-doubling or fine structure.

## Conclusions

In this article the treatment of interference between a bound state and a dissociative state based on the perturbative approach proposed by Fano^8^ is presented. The method was applied to a problem reported by Child and Lefebvre,^16^ showing excellent agreement. Subsequently, this procedure was applied to the case of the interaction between C ″^5^Π_*u*_ and 1 ^7^Σ_*u*_^+^ electronic states, where the potential energy curves of both states had already been calculated, as well as the spin–orbit coupling curve between them. In this way, it was possible to calculate the energy shifts Δ­(*E*_v_) for the bound energy levels, the squared Franck–Condon factors |⟨ϕ_E_||ψ_v_⟩|^2^ of the interaction between the electronic states as well as the broadening Γ of the vibrational levels.

At the same time, calculations of the radiative lifetimes of the transition between the electronic states C ″^5^Π_*u*_ → A ^′5^Σ_*g*_^+^ and the predissociative lifetimes of the C ″^5^Π_*u*_ electronic state, due to its interaction with the dissociative electronic state 1 ^7^Σ_*u*_^+^, were carried out using the LEVEL 16 ^21^ and BCONT 2.2 ^22^ programs, respectively. These calculations enabled the determination of dissociation rates and the effective lifetimes of the vibrational levels of the C ″^5^Π_*u*_ electronic state. The values obtained fully agree with the estimates derived from the calculation of the Γ broadening obtained using perturbative calculations.

Unfortunately, the absence of experimental data prevents a direct comparison with the results obtained in this work. We believe that further experimental efforts aimed at obtaining more information about the predissociation processes of the C ″^5^Π_*u*_ state are necessary.
